# Biot-Granier Sensor: A Novel Strategy to Measuring Sap Flow in Trees

**DOI:** 10.3390/s20123538

**Published:** 2020-06-22

**Authors:** Jucilene M. Siqueira, Teresa A. Paço, José Machado da Silva, José C. Silvestre

**Affiliations:** 1 LEAF—Linking Landscape, Environment, Agriculture and Food, Instituto Superior de Agronomia, Universidade de Lisboa, 1349-017 Lisboa, Portugal; tapaco@isa.ulisboa.pt; 2 DCEB—Departamento de Ciências e Engenharia de Biossistemas, Instituto Superior de Agronomia, Universidade de Lisboa, 1349-017 Lisboa, Portugal; 3 INESC TEC and Faculty of Engineering, University of Porto, 4200-465 Porto, Portugal; jms@fe.up.pt; 4 Instituto Nacional de Investigação Agrária e Veterinária, 2565-191 Dois Portos, Portugal; jose.silvestre@iniav.pt

**Keywords:** sap flow measurement, Granier sap flow index, low-cost sensors, sap flow data processing, heat dissipation

## Abstract

The Biot-Granier (Gbt) is a new thermal dissipation-based sap flow measurement methodology, comprising sensors, data management and automatic data processing. It relies on the conventional Granier (Gcv) methodology upgraded with a modified Granier sensor set, as well as on an algorithm to measure the absolute temperatures in the two observation points and perform the Biot number approach. The work described herein addresses the construction details of the Gbt sensors and the characterization of the overall performance of the Gbt method after comparison with a commercial sap flow sensor and independent data (i.e., volumetric water content, vapor pressure deficit and eddy covariance technique). Its performance was evaluated in three trials: potted olive trees in a greenhouse and two vineyards. The trial with olive trees in a greenhouse showed that the transpiration measures provided by the Gbt sensors showed better agreement with the gravimetric approach, compared to those provided by the Gcv sensors. These tended to overestimate sap flow rates as much as 4 times, while Gbt sensors overestimated gravimetric values 1.5 times. The adjustments based on the Biot equations obtained with Gbt sensors contribute to reduce the overestimates yielded by the conventional approach. On the other hand, the heating capacity of the Gbt sensor provided a minimum of around 7 °C and maximum about 9 °C, contrasting with a minimum around 6 °C and a maximum of 12 °C given by the Gcv sensors. The positioning of the temperature sensor on the tip of the sap flow needle proposed in the Gbt sensors, closer to the sap measurement spot, allow to capture sap induced temperature variations more accurately. This explains the higher resolution and sensitivity of the Gbt sensor. Overall, the alternative Biot approach showed a significant improvement in sap flow estimations, contributing to adjust the Granier sap flow index, a vulnerability of that methodology.

## 1. Introduction

The current availability and confluence of embedded and real time systems with wireless, small size sensors and networking technologies have allowed developing promising data capture and transmission infrastructures suitable for a technical, economic, and social revolution on solving crop irrigation issues [1]. In these infrastructures, sensors and communication networks are being meaningfully used in irrigation, fertilisation, horticulture, greenhouse, farming and monitoring of livestock and pastures [2]. However, the accurate characterisation of variables that influence the evapotranspiration (ET) measurement is often confined to the use of general purpose hardware and software modules intended for data acquisition and data processing, respectively [3]. In [4], the authors detailed that there is a real need for innovative methods that can provide sound and affordable estimates of ET, using “plug-and-play” features that link better with the farm managerial skills. The availability of flexible and low-cost sensors can help to overcome these constraints, with ET field information provided under parameterised and in real time adjusted local conditions. That is the motivation behind the development of the low-cost sap flow sensors proposed here, which ease the estimation of plants transpiration (T).

The wide applications of sap flow measurements on, e.g., crop evapotranspiration, plant water use, forest management or plant physiology, make it a significant technique to be taken in consideration in order to adequate water use for irrigation with efficient and productive strategies. The majority of the techniques currently available to estimate T at orchards and vineyards rely on the installation of sap flow sensors in the trunks, using heat as a sap flow tracer, since the process is limited by a single route of water extraction along the stem [5]. In [6], the authors summarised these approaches in heat balance, heat pulse and constant heater methods, which are fundamentally different in their operating principles [7,8], namely concerning the sensors placement in relation to the conductive organ, the sap flow calculation methods, the size of the stems, and the measurements frequency.

Currently, two common commercially available methods are used for sap flow quantification [6] that are adequate to use in woody plants [9]; the Granier heat dissipation method [10,11] and the heat pulse-sap velocity method [12]. Given the recognised replicability and automation of these sap flow approaches, they have been widely used mainly by woody stems to quantify tree transpiration [13]. However, the well-known Granier heat dissipation method is the most widely applied sap flux density method because of its simplicity and low cost [8]. Low cost derives from the fact that the technical details of these sensors are under public domain, in association with the low cost materials that make up these sensors.

The Granier heat dissipation method has been widely used for larger stems and has been reported as giving moderate to reasonable results, for example with olive trees [14,15], Mediterranean evergreen oak savannah [16], date palms trees [17], cherries orchards [18], vineyards [19,20]. However, some limitations have been observed in using the Granier approach. In [21], the authors detected errors associated with scaling single tree estimates and measurement errors associated with ring-porous water-conducting elements. Moreover, it was observed an underestimation of T with the Granier approach data, compared to data obtained with the eddy covariance (EC) micrometeorological method [22]. In [23], the authors suggested that the original Granier calibration should not be assumed for all cases. Also, it was observed that sap flow estimates with young olive trees varied with sensors’ position [24]. Other limitations may be pointed out to the method, such as the poor performance for near null fluxes, the inability to measure reverse flows, the increased wounding effect due to continuous heating and the natural temperature gradients [8].

Some studies have aimed to improve the Granier methodology. For example, in [25] the authors proposed a new equation for improving the measurement accuracy within an error of about 4%. The proposed approach appeared to be an easier solution compared to potted trees and particularly suitable for orchards, thus contributing to improve irrigation management worldwide. In [26], the authors presented a new modification of the Granier sap flow method that keeps the temperature difference of the needle pair constant, by adjusting the heating power to the upper needle, thus improving its temporal resolution. The method further reduces the power requirements of the sap flow measurements but does not solve the general over or underestimation of sap flux density at certain conditions. Other authors [27] have proposed a cyclic heat dissipation method offering a solution to the natural thermal gradient (NTG) problem, by means of the extrapolation of a cyclic power schema (CPS) of data acquisition, as well as providing a better opportunity of understanding the mechanism of the NTG bias.

The advantages of the Granier method, as an alternative to other sap flow sensing approaches, appear to be the unproblematic installation, low-cost and low demanding in processing and interpreting the collected data [28]. Taking advantage of these benefits, one justifies the upgrading of Granier methods to minimise their constraints.

The conventional Granier heat dissipation methodology recurs to tree transpiration evaluation made after heating a spot slightly inside the tree trunk (in the xylem) by means of a heating element and measuring the temperature variation observed in a spot 10 cm below that heating spot. Transpiration is inferred after relating the heat transfer with the sap flow. Typically, two thermocouples are used to measure that temperature difference (∆T, °C).

In the present study, the conventional Granier (Gcv) methodology was upgraded to measure the absolute temperatures in the two observed spots, what allows to perform the Biot number approach, hence naming this approach as Biot-Granier approach (Gbt). The measurement of the two temperatures, instead of the temperature difference between them, provides favourable conditions for a better understanding of the thermodynamics that involves this technique and facilitates the development of mathematical procedures that can assist in more accurate measurements. In addition, the changes in the sensors will not affect their simple manufacturing and low cost. 

Hence, the work presented herein addresses the evaluation of the Biot-Granier sensors, concerning technical and computational performance, after comparison with a commercial sap flow sensing device and independent data (i.e., eddy covariance technique, vapour pressure deficit and volumetric water content). The performance of the new sap flow Gbt sensor was evaluated in three short-term preliminary trials: potted olive trees in a greenhouse and two vineyards (varieties “Tempranillo” and “Galego Dourado”). 

## 2. Materials and Methods

As for the theoretical reasoning, this work considers the use of two sensors to measure the temperatures at the heated $\left(T_{\mathrm{heat}},{}^{\circ}C\right)$ and the unheated observation points $\left(T_{\mathrm{no}-\mathrm{heat}},{}^{\circ}C\right)$, from which ΔT(°C) is computed as:

(1) $$\Delta T=T_{\mathrm{heat}}-T_{\mathrm{no}-\mathrm{heat}}$$

Conventionally, the Granier sap flow index ($k_{\mathrm{cv}}$, dimensionless) is obtained with the empirical Granier equation:

(2) $$k_{cv}=\frac{\left(\Delta T_{MAXcv}-\Delta T\right)}{\Delta T},$$

where $\Delta T_{MAXcv}\;\left({}^{\circ}C\right)$ is the static ΔT maximum value on a daily course, which is related to the minimum or null sap flow rate for that specific day. Thus, the term $T_{MAXcv}$ is computed as:

(3) $$T_{MAXcv}=\Delta T_{MAXcv}+T_{\mathrm{no}-\mathrm{heat}}$$

The conventional sap flux density ($u_{cv}$, m^3^ m^−2^ s^−1^) is calculated from $k_{cv}$ using a relationship admitted to being species independent [10,11,29] and based on the *Granier* calibration equation (Equation (4)):

(4) $$u_{cv}=118.99\times 10^{-6\;}k_{cv}{}^{1.231}$$

The conventional sap flow (Fcv, m^3^ s^−1^) can be calculated as:

(5) $$F_{cv}=u_{cv}\times A$$

where A (m^2^) is the area of the conducting xylem section.

The term $\Delta T_{MAXcv}$ is intangible and supposed to be the temperature when the sap is quiet or in minimum motion and dependent on the term $T_{\mathrm{no}-\mathrm{heat}}$ measured with the second sensor. The determination of these values is critical since they affect the complete raw sap flow data [11,29]. Therefore, the $T_{\mathrm{heat}}$ gauge should be adjusted to the $T_{\mathrm{no}-\mathrm{heat}}$ gauge to estimate the correct ΔT maximum value and the actual environment temperature $\left(T_{\infty }\right)$ terms.

Equation (2) was written assuming that the transient cooling process takes place in the interim period before equilibrium is established. Consequently, term $k_{cv}$ would represent the ratio of heat transfer by convection to the heat transfer by conduction. In fluid dynamics, this ratio is called the Biot number [30], and it is selected to identify transient conduction problems when convection is the rate-controlling process. 

Accordingly, the functional form of the sap thermal dynamics around the heater describes heat transfer in the coupled convective and conductive heat transfer situation, imputing physical meaning to the Biot number. Hence, the limiting steady-state temperature is distributed in three measurements, i.e., the maximum reached temperature with null sap flow $\left(T_{MAX},{}^{\circ}C\right)$, local sap reached temperature $\left(T_{\mathrm{heat}},{}^{\circ}C\right)$ and environment temperature $\left(T_{\infty },{}^{\circ}C\right)$. $T_{\mathrm{heat}}$ is the sap temperature departing from the environment temperature $\left(T_{\infty },{}^{\circ}C\right)$ and thus fluctuating for some measure intermediate, being $T_{\infty }<T_{\mathrm{heat}}<T_{MAX}$. One can find the Biot number approximation and its dependency (i.e., $T_{MAX}$ and $T_{\infty }$ estimates) on the geometry and thermal properties of the system and on the flow rate regime. 

### 2.1. Biot-Granier Algorithm

The Biot-Granier algorithm is a crucial component to set up the Biot-Granier sensing approach (Gbt), including data management and automatic data processing. The latter refers to the non-compulsory automated algorithms to process raw data using relatively simple procedures, e.g., the Fuzzy Algorithm Automation System (FAUSY) [31]. 

Termed after the analogy with the Biot number, the Biot-Granier sap flow index $(k_{bt}$, dimensionless) is computed from the linear regression curve of the terms $T_{\mathrm{heat}}$ and $T_{\mathrm{no}-\mathrm{heat}}$ on the night-time period, considering that during this period sap flow is null, and thus, the $T_{\mathrm{heat}}$ term is equal to the $T_{MAX}$ term. A linear regression equation (Equation (6)) can be used to characterise the pair $T_{\mathrm{heat}}$ and $T_{\mathrm{no}-\mathrm{heat}}$ individually, working as a factor of adjustment relating the measurements $T_{\mathrm{heat}}$ and $T_{\mathrm{no}-\mathrm{heat}}$ to the terms $T_{MAX}$ and $T_{\infty }$:

(6) $$T_{MAX}=T_{\mathrm{heat}\left(null\;sap\;flow\right)}\to T_{MAX}=a\times T_{\mathrm{no}-\mathrm{heat}}+b$$

where *a* is the slope of the regression curve and *b* is the $\Delta T_{MAX}$ term. Also, the environment temperature $\left(T_{\infty }\right)$ is in principle defined when $\Delta T_{MAX}$ is null. Thus:

(7) $$\Delta T_{MAX}=0\;\to \;T_{\infty }=a\times T_{\mathrm{no}-\mathrm{heat}}$$

and the Biot-Granier sap flow index $\left(k_{bt}\right)$ is solved as:

(8) $$k_{bt}=\frac{T_{MAX}-T_{\mathrm{heat}}}{T_{\mathrm{heat}}-T_{\infty }}\;\;$$

**Figure 1:**
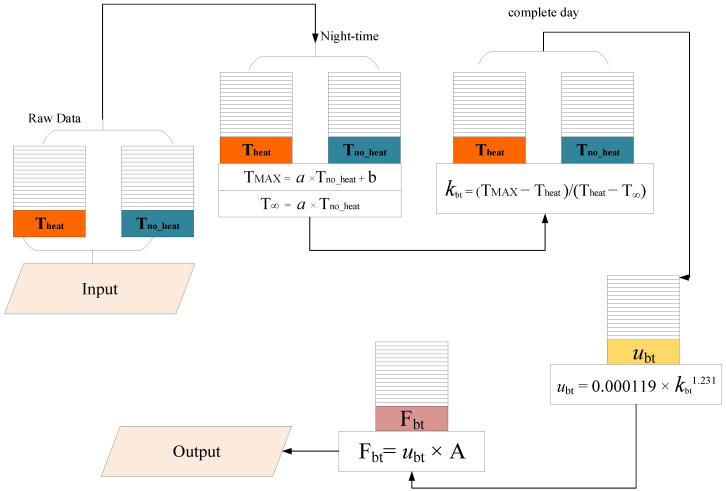
Transpiration computation flow. $T_{\mathrm{heat}}$ (local heated sap temperature); $T_{\mathrm{no}-\mathrm{heat}}$ (local unheated sap temperature); $T_{\mathrm{MAX}}$ (maximum sap temperature); $T_{\infty }$ (environment temperature); $k_{bt}$ (Biot-Granier sap flow index); $u_{bt}$ (Biot-Granier sap flow density); Fbt (transpiration with Biot-Granier approach).

Figure 1 shows the data processing flow used to convert the measured data values $(T_{\mathrm{heat}}$ and $T_{\mathrm{no}-\mathrm{heat}})$ into transpiration estimates.

For comparison purposes, the Granier sap flux density ($u_{cv})$ is computed as the conventional Granier sap flow index $\left(k_{cv}\right)$, in which it extracts the $\Delta T_{MAX}$ values directly from the measured raw data. Thus, $k_{cv}$ is computed based on Equation (2) and reformulated as:

(9) $$k_{cv}=\frac{\Delta T_{MAXcv}+T_{\mathrm{no}-\mathrm{heat}}-T_{\mathrm{heat}}}{T_{\mathrm{heat}}-T_{\mathrm{no}-\mathrm{heat}}}$$

### 2.2. Biot-Granier Sensor 

**Figure 2:**
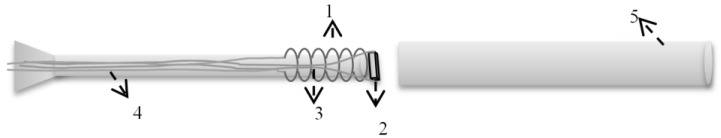
Biot-Granier sensor design. Building components: 1. nickel-chrome wire; 2. Thermistor; 3. Thermistor terminals; 4. Hypodermic needle; 5. Stainless tube.

According to the assembling technique demonstrated in [32], Figure 2 illustrates the Gbt sensor assembly parts. The Gbt device functionality relies on two operations: temperature sensing and heating. Temperature sensing is performed with a thermistor inserted in a glass micropipette and glued into place inside this micropipette using a cyanoacrylate adhesive. The location of the thermistor in the micropipette determines the depth at which temperature measurements can be taken inside the trunk tree. Heating is achieved after the Joule heating effect within a nickel-chrome wire. Only one of the sensing devices includes the heater.

**Figure 3:**
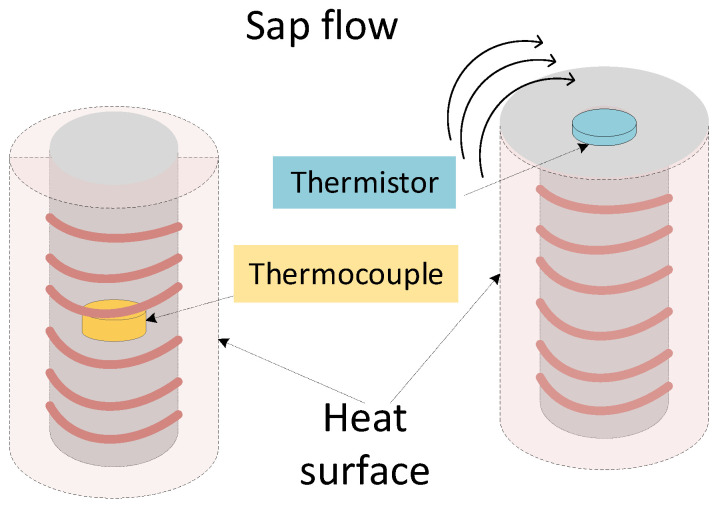
Building differences between Biot-Granier sensors (**right**) and conventional sensors (**left**).

The fundamental difference between Gbt and Gcv approaches resides in the fact that Gcv only provides a temperature difference measurement, while the system being proposed here provides also the two absolute temperatures, allowing for the Biot-Granier implementation, as well as for a more accurate observation process. Another core difference is the placement of the temperature sensing device in the body of the sensor. Gcv sensors place the temperature sensing device in the middle of the heating component, while in the Gbt ones it is placed in the tip of the needle to be inserted in the trunk (Figure 3). This allows the Gbt sensors to measure the temperature changes on the spot affected by sap motion. In the conventional Granier method the sensor provides the integral of the temperatures in the cylinder around the needle. With the modification being proposed, it is expected that the sensitivity to these temperature fluctuations is improved. Table 1 summarises the differences between Gbt and Gcv designs.

### 2.3. Experimental Setup

The Gbt sensor sets were tested in three experiments, where Experiment I was carried out in a greenhouse and the other two were set in experimental open fields. The raw data obtained from these trials were compared and evaluated using bias analysis and graphical techniques. 

#### 2.3.1. Experiment I

Experiment I was carried out from 10 March to 20 April 2018 in a greenhouse located at the Instituto Nacional de Investigação Agrária e Veterinária (INIAV-Dois Portos, Portugal). Four potted (capacity of 25 L) olive trees (P01, P02, C01 and C02) were used in this study, which contained a medium consisting of 50% of hummus and 50% of perlite. Table 2 describes the olive trees, i.e., trunk area, azimuth, position.

The potted tree surfaces were covered with cling film to stop evaporation from the medium surface. The sensor pairs and the olive trunk were covered with a thick aluminium foil to limit the effects of sunlight on trunk temperature. Each sensor set was implanted into the olive trunk to a depth of 20 mm, using a 2.0 mm diameter drill bit. The two sensor sets were separated by a vertical distance of at least 10 cm. The Gbt sensor sets (P01 and P02) were monitored using the measurement of sap flux density $\left(u_{cv},m^{3}\;m^{-2}\;s^{-1}\right)$ of two commercially manufactured Granier sensor sets (C01 and C02; UP GmbH, Ibbenbüren, Germany). The Gbt sensor sets (P01 and P02) data were recorded with 15-min measurement intervals and scaled to the hourly mean values, according to the analysis required.

A gravimetric test was carried out for the potted trees P01, P02, C01 and C02, one at a time, which were transferred onto a weighing platform with 30 kg capacity and 0.5 g accuracy (Radwag-WLC 30/F1/R, RADWAG, Radom, Poland). The potted trees were irrigated during the previous 24 h to ensure the same hydric conditions between treatments. The sap flux density rates $\left(u_{Grav},m^{3}\;m^{-2}\;s^{-1}\right)$ were obtained by weighing the potted olive trees every 30 s and averaging measures to 10-min intervals by means of a data logger and scaled to the hourly mean values, according to the analysis required. 

Linear regressions (Equation (6)) were computed to characterise the Gbt sensor sets (P01 and P02) individually, working as a factor of adjustment relating the measurements $T_{\mathrm{heat}}$ and $T_{\mathrm{no}-\mathrm{heat}}$ to the terms $T_{MAX}$ and $T_{\infty }$.

For comparative purposes, the hourly mean values of the air vapour pressure deficit (VPD, kPa) estimates were collected from 10 March to 20 April 2018, at INIAV-Dois Portos meteorological station, at 150 m from the vineyard.

#### 2.3.2. Experiment II

Experiment II was completed in a vineyard with the variety ‘Tempranillo’, located at Lezíria do Tejo wine region (Adega Catapereiro, EN118 Porto Alto, Alcochete, Portugal). The experimental plot is part of a large vineyard of approximately 100 ha, resulting in an annual average wine production of 612,000 L. The mean height tree from ground is about 1.4 m, planted with a density of 3300 plants/ha (3.0 × 1.0 m).

It was carried out with one pair of Biot-Granier sensors (SP04) from 20 July to 28 August 2017. A commercial Granier sensor set (Gr3; UP GmbH) was also used for comparison purposes. The diameters at the breast height are 22 cm^2^ (SP04) and 13 cm^2^ (Gr3) and the sapwood depth is estimated as being close to diameter at breast height (excluding bark depth). This experiment aimed at performing the comparison between Biot-Granier and conventional Granier approaches and to analyse the physical phenomena of heat transfer between the heater sensor and sap. The temperature difference data (ΔT,°C) from SP04 and Cr3 sensor sets were recorded in a data logger (Model CR1000, Campbell Scientific, Inc., Logan, UT, USA), obtained with 15-min measurement intervals and scaled to the hourly mean.

#### 2.3.3. Experiment III

Another vineyard study occurred between 1 September to 31 October 2016 in a grapevine belonging to the demarcated wine region of Carcavelos of the variety “Galego Dourado”. The vineyard is located at the Estação Agronómica Nacional—Quinta do Marquês, Oeiras, Portugal. The experimental plot is part of a large vineyard of 12.5 ha. The plant density is 3336 plants/ha, and the plantation spacing of the crop is 2.5 m per 1.1 m. The vineyard was not irrigated, but rainfall occurred during the study. The raw data in rainfall occurrences were not considered since eddy covariance sensors do not work accurately during such periods. The direct measurement of evapotranspiration was carried out using the eddy covariance technique. The sensors were placed in a metal observation tower at the height of 2.5 m (at about twice the mean tree height from ground) oriented towards the prevailing winds, over a parcel of about 2.4 ha. Therefore, it would be necessary to ensure a fetch, in the direction of prevailing winds, equal to or higher than 200 m. A footprint analysis was performed based on [33]. The sensible and the latent heat flux $\left(W,m^{-2}\right)$ measurements were obtained with wind speed and temperature fluctuations measured by a three-dimensional sonic anemometer (CSAT3-3D, Campbell Scientific). The water vapour concentration was measured with an open path, infrared absorption gas analyser (IRGA) (LI7500, LiCor Inc., Lincoln, NE, USA). The eddy covariance data was configured to obtain 15-min mean fluxes. 

A pair of Biot-Granier sensors (SP32L) was installed to estimate the transpiration. The diameter at the breast height was 8 cm^2^ (SP32L) and the sapwood depth was estimated as being close to diameter at breast height. The Siqueira methodology [34]—a weighing device (mLy) and one pair of calibrated Peltier cells (Pcell) were buried in the row, positioned midway between trees (under-canopy)—was used to estimate the soil moisture, the soil heat flux and the soil heat storage. 

Table 3 summarizes the description of the three experiments.

## 3. Results and Discussion

### 3.1. Experiment I

#### 3.1.1. Evaluation of Sensors with the Biot-Granier Sap Flow Index Approach

In Experiment I, the Biot-Granier sap flow index $\left(k_{bt}\right)$ was analysed regarding the adjustment of $T_{MAX}\;\left({}^{\circ}C\right)$ and $T_{\mathrm{no}-\mathrm{heat}}\;\left({}^{\circ}C\right)$. The $k_{bt}$ term was computed after the linear regression curve (lrc) obtained between two terms: $T_{\mathrm{heat}}$ (°C) and $T_{\mathrm{no}-\mathrm{heat}}$ (°C) registered at night-time. $T_{MAX}$ (°C) and $T_{\infty }$(°C) are computed with Equations (10)–(13) achieved with the lrc obtained from the Gbt sensor sets P01 and P02, respectively:

(10) $$T_{MAX\left(P01\right)}=0.9540\times T_{\mathrm{no}-\mathrm{heat}\left(P01\right)}+4.5088\left(R^{2}=0.9996\right)\;,$$

(11) $$T_{MAX\left(P02\right)}=1.0414\times T_{\mathrm{no}-\mathrm{heat}\left(P02\right)}+7.2937\left(R^{2}=0.9996\right)\;\;,$$

(12) $$T_{\infty }=0.9540\times T_{\mathrm{no}-\mathrm{heat}\left(P01\right)}\;,$$

(13) $$T_{\infty }=1.0414\times T_{\mathrm{no}-\mathrm{heat}\left(P02\right)}\;,$$

The computation of the maximum temperature with the Biot-Granier (Equation (6)) and conventional (Equation (3)) approaches are configured as dynamic and static, respectively.

**Figure 4:**
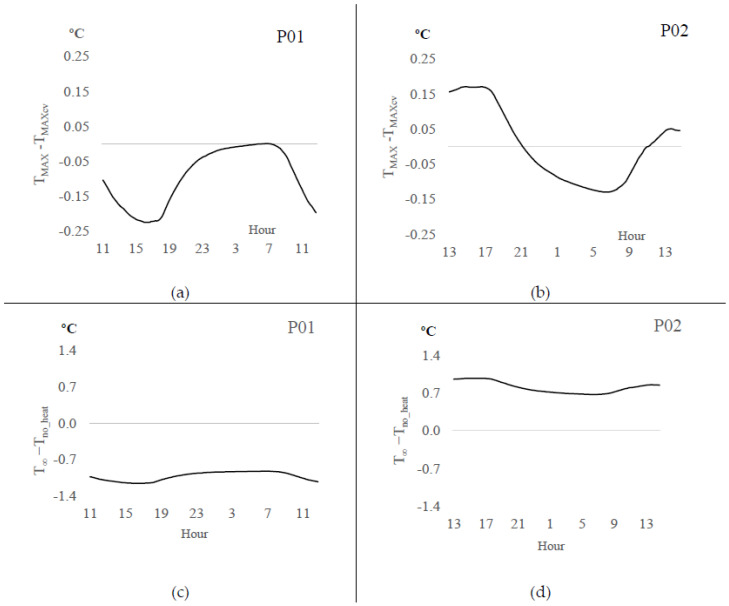
Comparison between Biot-Granier and conventional *Granier* approaches (Experiment I). (**a**) offset between T_MAX_ and T_MAXcv_ obtained with the sensor T_heat(P01)_; (**b**) offset between T_MAX_ and T_MAXcv_ obtained with the sensor T_heat(P02)_; (**c**) offset between T_no-heat_ and T_∞_ obtained with the sensor T_no_heat(P01)_; (**d**) offset between T_no-heat_ and T_∞_ obtained with the sensor T_no_heat(P02)_.

Figure 4a–d show the adjustment magnitude obtained with the Biot-Granier approach ($T_{MAX})$, compared to the conventional approach $\left(T_{MAXcv}\right)$. The offset $T_{MAX}-T_{MAXcv}$ from sensor P01 ranged between 0 °C and −0.22 °C (Figure 4a). Conversely, sensor P02 showed an offset $T_{MAX}-T_{MAXcv}$ (Equations (11) and (2)) alternating between −0.16 °C and +0.16 °C, capturing positive bias during the time interval 13:00 to 21:00 h and inverting to negative bias during the time from 21:00 to 13:00 h (Figure 4b). Also, Figure 4c,d show the $T_{\infty }$ adjustments, wherein the bias between $T_{\infty }$ (Equations (12) and (13)) and $T_{\mathrm{no}-\mathrm{heat}}$ obtained with both sensors P01 and P02 had performed differently. Sensor set P01 (Figure 4c) showed smaller $T_{\infty }$ values than $T_{\mathrm{no}-\mathrm{heat}}$, and sensor set P02 (Figure 4d) reached higher $T_{\infty }$ values than $T_{\mathrm{no}-\mathrm{heat}}$. Given that the different approaches provide different ∆T (Equations (1) and (7)) and consequently, the maximum different temperatures, thus, it integrates differently the thermal effects when surrounded with sap. 

The validation of the Biot-Granier approach was performed with the gravimetric test, aiming to evaluate the agreement with the actual sap flux density estimates ($u_{Grav}$, m^3^ m^−2^ s^−1^) and the relevance of adjustments referred in Figure 4a–d. The sap flux density rates were calculated from $k_{cv}$ and $k_{bt}$, respectively, $u_{cv}$ (m^3^ m^−2^ s^−1^) and $u_{bt}$ (m^3^ m^−2^ s^−1^), and were compared with $u_{Grav}$, using the sensor sets P01 and P02.

**Figure 5:**
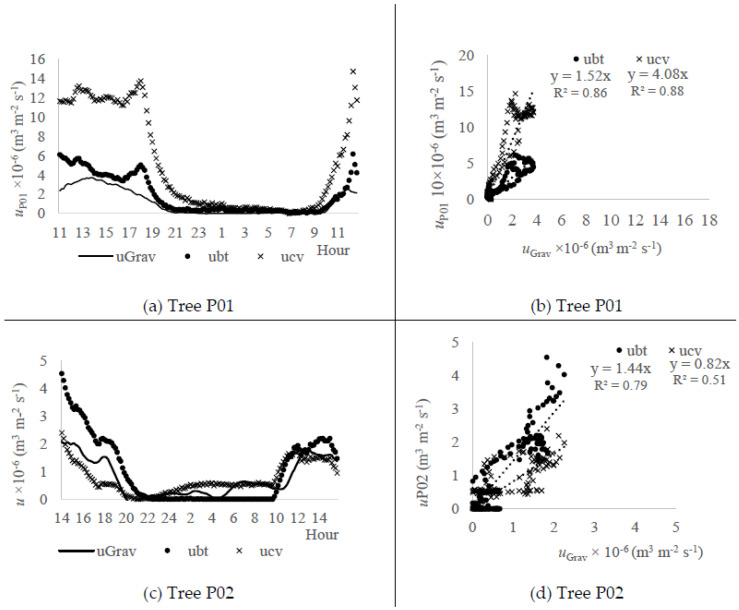
Values of sap flux density computed after the Biot approach (*u_bt_*, m^3^ m^−2^ s^−1^) and the conventional Granier approach (*u_cv_*, m^3^ m^−2^ s^−1^) in comparison with the sap flux density observed with the gravimetric test (*u_Grav_*, m^3^ m^−2^ s^−1^) on the sensor (Experiment I): (**a**) P01, circadian curves (26–27 March 2018); (**b**) P01, regression curve *u_Grav_* versus *u_bt_* and *u_cv_*; (**c**) P02, circadian curves (27–28 March 2018); (**d**) P02 regression curve *u_Grav_* versus *u_bt_* and *u_cv_*.

Figure 5a shows the circadian curves obtained with $u_{bt}$ and $u_{cv}$ data (sensor P01) in comparison to $u_{Grav}$ data. The best agreement occurred within $u_{bt}$ values, given that $u_{cv}$ data overestimated the sap flow rate 4 times when compared to $u_{Grav}$ (Figure 5b). Nevertheless, $u_{bt}$ showed values that estimated as 1.5 times the $u_{Grav}$ values. The adjustments based on the Biot equations in sensor P01 (Equations (10) and (12)) contributed to a reduction of 2.7 times on the overestimate obtained with the conventional approach, whose offset magnitudes were estimated as −0.2 °C and −1.2 °C for $T_{MAX\left(P01\right)}$ and $T_{\mathrm{no}-\mathrm{heat}\left(P01\right)}$ data, respectively. Also, both $u_{bt}$ and $u_{cv}$ estimates presented relative correlation (Figure 4b), showing that the sap flow estimates were qualitatively compatible, which is a useful information for sensors calibration.

Figure 5c shows the circadian curve obtained with $u_{bt}$ and $u_{cv}$ data (sensor P02) in comparison to $u_{Grav}$ data. It was observed that the best agreement occurred with $u_{bt}$ values, being qualitatively compatible (Figure 5d), since the sap flux density ($u_{bt}$) from P02 sensor set overestimates 1.44 times, when compared to $u_{Grav}$. Nevertheless, $k_{cv}$ showed values closer to the $u_{Grav}$ data range, underestimating 0.82 times the $u_{Grav}$ values, albeit presenting a weak correlation compared to $u_{Grav}$ measured from gravimetric approach (Figure 5d).

The low sap flow rates observed within P02 potted tree (trunk area: 18 cm^2^) compared to the P01 potted tree (trunk area: 24 cm^2^) are plausible, given the smaller trunk area. Also, it is verified that $u_{bt}$ computed with the $k_{bt}$ approach reacted more often to the null sap flow in the night-time (Figure 5c). The same did not occur with $u_{cv}$ computed with the $k_{cv}$ approach.

**Figure 6:**
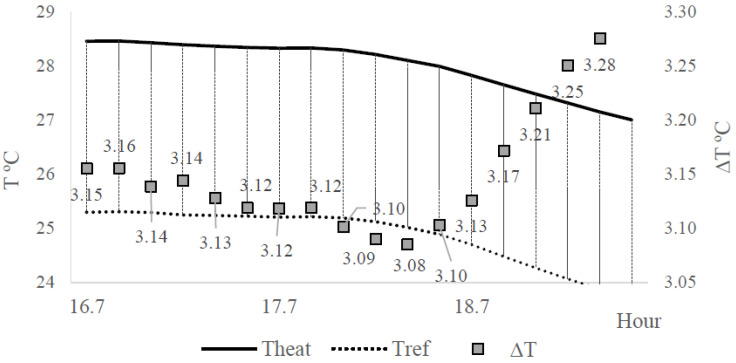
Peak observed in the sap flux density measurements (Experiment I). Left vertical axis: temperature (T, °C). Right vertical axis: temperature difference (∆T, °C).

Figure 5a illustrates a peak observed at hours 16 to 19 of the day that had been detached (Figure 6) for analysis. Figure 6 shows that ∆T values decreased, ranging initially from 3.16 to 3.08 °C, and at that point, increased up from 3.08 to 3.28 °C. This could be due to the dissonant thermal properties variation between $T_{\mathrm{heat}}$ and $T_{\mathrm{no}-\mathrm{heat}}$ sensor places, caused by the surrounding disturbance, e.g., natural thermal gradients (NTG), as mentioned by [29], as well as, motion influenced by the wood thermal properties surrounding $T_{\mathrm{heat}}$ G_bt_ sensor.

Sap flow estimates are considered challenging, mainly due to the empirical equation that transfers the temperature values to sap flux density being strongly sensitive to $\Delta T_{MAX}$ [35] and to the reference temperature being collected from another sensor. The main point here is that the $\Delta T_{MAX}$ values dynamically adjusted by the Biot-Granier approach demonstrated to be more appropriate than the conventional approach. The conventional configuration of these values (static $\Delta T_{MAXcv}$) might cause significant errors in the sap flow index computations. 

**Figure 7:**
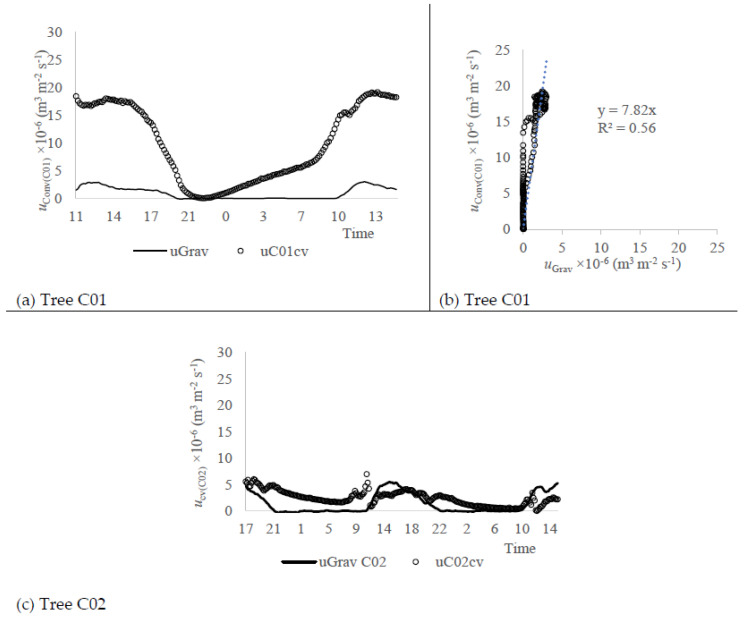
Sap flux density computed with the conventional Granier approach (*u_cv_*, m^3^ m^−3^ s^−1^) and that obtained with the gravimetric test (*u_Grav_*, m^3^ m^−3^ s^−1^) on the sensor (Experiment I): (**a**) C01, circadian curves (17–18 April 2018); (**b**) C01, regression curve *u_Grav_* versus *u_cv_*; (**c**) C02, circadian curves (18–20 April 2018).

The conventional sensors C01 and C02 were evaluated, respectively, with the gravimetric approach. Figure 7a shows the circadian curve obtained with $u_{cvC01}$ (m^3^ m^−2^ s^−1^) in comparison to $u_{Grav}$ (m^3^ m^−2^ s^−1^) data. The conventional approach was used to compute the Granier sap flow index ($k_{cv}$), which overestimated it as 7.82 times and presented a weak correlation compared to the $u_{Grav}$ measured with the gravimetric approach (Figure 7b). Figure 7c shows the circadian curve and linear regression obtained with $u_{cvC02}$ (m^3^ m^−2^ s^−1^) in comparison with the $u_{Grav}$ (m^3^ m^−2^ s^−1^) data. The $u_{cvC02}$ values show to be compatible in magnitude to the $u_{Grav}$ values range but present no correlation with $u_{Grav}$ measured with the gravimetric approach.

According to [29], $T_{MAX}$ should be determined separately for each sensor because $T_{MAX}$ is a sensor-specific result. This can be due to variations occurred when manufacturing and installing each one of the sensors (e.g., namely the effective resistance of the heater). In a better way, the $k_{bt}$ approach reduced the bias between $T_{\mathrm{heat}}$ and $T_{\mathrm{no}-\mathrm{heat}}$ sensors, as well, encompassed the characteristics of the conventional daily $\Delta T_{MAX}$ determination and upgraded to a stable Δ*T* computation in the night-time, obtaining Δ*T* with zero sap flow conditions, i.e., $\Delta T_{MAX}$ from G_bt_ sensors. This is made possible because G_bt_ sensors measure absolute temperatures. Thus, based on the analyses of sap flow data of P01 and P02 olive trees, this study showed that $T_{MAX}$ and $T_{\infty }$ adjustments based on the Biot approach provide the best estimates, allowing for a robust, physically-based $\Delta T_{MAX}$ determination, reliable absolute sap flux density computations and with the ability of self-regulating under nocturnal sap flow. 

#### 3.1.2. Comparison between Biot-Granier and Conventional Granier Sensors

**Figure 8:**
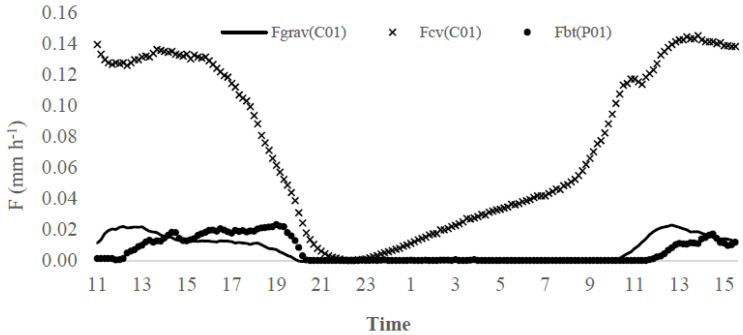
Sap flow measured in potted olive tree (P01) and computed with the Biot-Granier approach (F_bt(P01)_, mm h^−1^), and the sap flow observed for gravimetric test (F_grav(C01)_, mm h^−1^) in potted olive tree (C01) and computed for conventional Granier approach (F_cv(C01)_, mm h^−1^) in days 17–18 April 2018 (Experiment I).

Sap flow measurements (F, mm h^−1^) obtained with sensor sets P01 and C01 applied in potted trees with similar dimensions, were used for comparison purposes. Figure 8 illustrates sap flow estimates obtained with the gravimetric (*F*_grav(C01)_, mm h^−1^) and the conventional (*F*_cv(C01)_, mm h^−1^) approaches, using sensor C01, and the Biot-Granier (*F*_bt(P01)_, mm h^−1^) approach, with sensor P01. Albeit the gravimetric data (*F*_grav(C01)_) were originated from the C01 olive tree, the *F*_grav(C01)_ data magnitude showed to be more similar to the *F*_bt(P01)_ data from the P01 olive tree than *F*_cv(C01)_ data. The *F*_cv(C01)_ data inconsistency is due to conventional sap flow index ($k_{cv}$) computation, where the $k_{cv}$ equation terms were not adequately adjusted. Indeed, the ΔT_MAX(C01)_ was higher 3 °C in the gravimetric test (days 17–18 April 2018) than in previous days. Unfortunately, it is not possible to compute the C01 sap flow index via the Biot approach in conventional Granier sap flow sensors, as they do not provide absolute temperature values.

**Figure 9:**
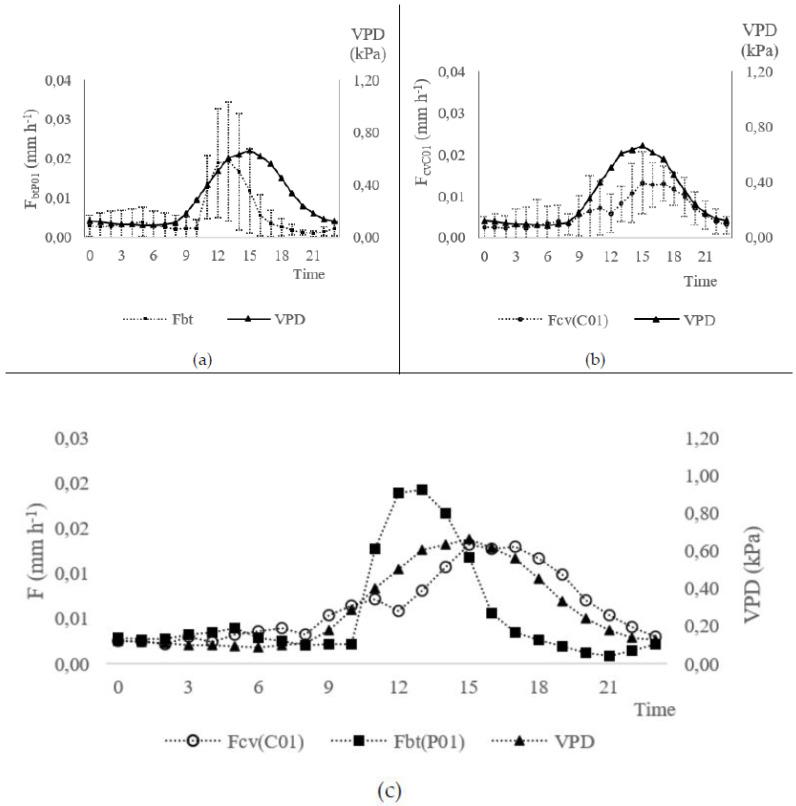
Hourly mean air vapour pressure deficit (VPD, kPa) from data collected at INIAV-Dois Portos meteorological station (days 27 March–18 April 2018) with (Experiment I): (**a**) sap flow from Biot-Granier sensors (F_bt(P01)_, mm h^−1^); (**b**) sap flow from the conventional *Granier* sensor (F_cv(C01)_, mm h^−1^); (**c**) Comparison between F_bt(P01)_, F_cv(C01)_ and VPD estimates.

Figure 9a–c show the hourly mean values of the transpiration obtained with sensors P01(*F*_bt(P01)_, mm h^−1^) and C01(*F*_cv(C01)_, mm h^−1^) in comparison with the hourly mean values of the air vapour pressure deficit (VPD, kPa) estimates from data collected from 27 March to 18 April 2018. *F*_bt(P01)_, *F*_cv(C01)_ and VPD data were clustered on an hourly basis. The *F*_bt(P01)_ circadian curve is related to VPD estimates (Figure 9a). In fact, the low values of VPD (<0.66 kPa) are related to low sap flow rates, as it is verified in *F*_bt(P01)_. Also, *F*_cv(C01)_ (Figure 9b) presented satisfactory data consistency, when compared to the VPD circadian curve. 

The *F*_bt(P01)_ and *F*_cv(C01)_ data are similar within the magnitudes of their values (Figure 9c), what is contradictory with the previous conclusion in Figure 8, given that the averaged values *F*_cv(C01)_ minimize the error caused for the overestimated values occurred in the days 17–18 April 2018. Usually, errors of such magnitude are acceptable for practical applications in Agriculture. An error lower than 10% can be accepted in agricultural applications, as processes efficiency is normally in this range. For example, the efficiency of irrigation systems is generally lower than 90%. *F*_bt(P01)_ show very low sap flow values, especially between times 12:00 and 18:00 (local time) reaching to almost zero, which was possibly influenced by stomatal closure [36,37].

In contrast, F_cv(C01)_ does not present a similar sensitivity. The reason resides in the geometry of the G_bt_ sensor that permits measuring the actual temperature of the sap. As a result, it can be easier to detect the elevation of temperature from the $T_{\mathrm{heat}}$ sensor, which should be closer to $T_{Max}$ temperature estimated, thus, reducing $k_{bt}$.

### 3.2. Experiment II

**Figure 10:**
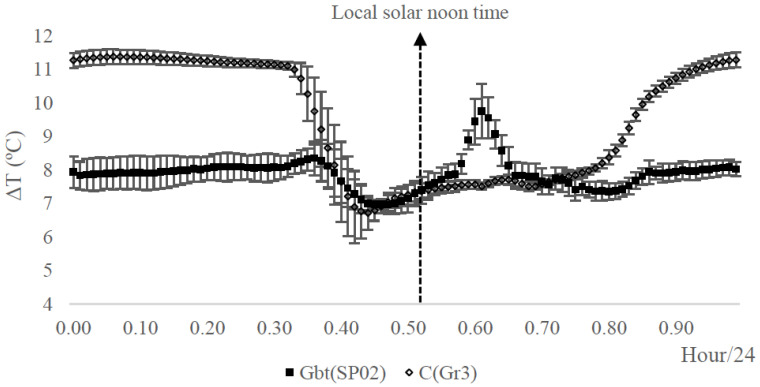
Temperature differences (ΔT, °C) collected with the sap flow sensors in the Adega Catapereiro Vineyard from day 20 to 28 August 2017. Experiment II: the hourly mean (ΔT, °C) with GbtSP02 and CGr3 sensors were clustered on an hourly basis (Hour/24). Full square—Biot-Granier data (ΔT (GbtSP02)). Empty square—conventional Granier data (ΔT (CGr3)).

In Experiment II, the range of differences observed between the maximum and minimum temperature is related to the capacity of the sensor of transmitting the heat dissipation caused by the sap flow. The G_btSP02_ sensor show lower heating capacity when compared with the C_Gr3_ sensor yielding lower ΔT values than the C_Gr3_ sensor (Figure 10). 

The heating capacity of the G_btSP02_ sensor provided a minimum of around 7 °C and maximum about 9 °C, contrasting with C_Gr3_ (minimum around 6 °C and a maximum of 12 °C). Probably, this is caused by operational features of G_bt_ sensors, as the position of thermistors (temperature sensing) in the tip of the sensor allow to capture more accurately the sap temperatures. Also, it explains the higher resolution of the G_btSP02_ sensor when compared with the C_Gr3_ sensor, corroborating with the high sensitivity of G_bt_ observed in the greenhouse trial.

A peak was observed systematically around 14:00 h, after the local solar noon time (at 12:40 h) (Figure 10). Possibly, this was caused by stomatal closure, which reduced transpiration, therefore increasing ΔT. This mechanic stomatal closure, occurring soon after solar noon, is known to occur in olive trees as referred in [38], especially under stress conditions and vineyard [39,40]. The vineyard was not being irrigated on the days from 20 July to 28 August 2017, and it is likely to admit some water stress. In comparison, it was not observed any peak in the C_Gr3_ sensor, given the low sensitivity to measuring the sap temperature.

### 3.3. Experiment III

In Experiment III the relation between Biot-Granier sensor estimates and independent data was evaluated. The vineyard considered in this experiment is cultivated under rainfed conditions. It is acceptable to consider the *ET_EC_* measurements as referring solely to the transpiration estimates during no rain periods, in summer.

**Figure 11:**
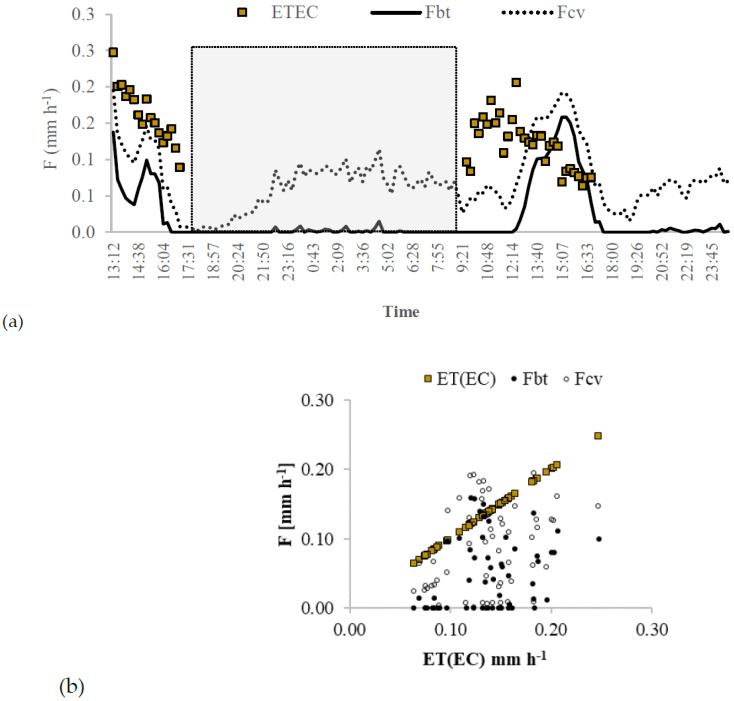
Raw data obtained between days 14–16 October 2016 with an eddy covariance instrument (EC) and a Biot-Granier sensor set (SP32L) (Experiment III): (**a**) circadian curve of the evapotranspiration measurements (ET(EC), mm h^−1^) and the adjusted sap flow rate measured in function of conventional Granier sap flow index (k*_cv_*) and Biot number Granier sap flow index (k_bt_) approaches; (**b**) dispersion of the transpiration data (F_bt_ and F_cv_) around the fitted line of the ET(EC).

Figure 11a shows the circadian curve of the *ET_EC_* comparing the conventional *Granier* sap flow index ($k_{cv}$) and the Biot-Granier sap flow index ($k_{bt}$) approaches, respectively. Figure 11b shows the dispersion of the transpiration obtained from G_btSP32L_ sensor data (*F_bt_* and *F_cv_*), around the fitted line of the *ET_EC_*. Albeit it is not verified a satisfactory correlation between transpiration obtained from G_btSP32L_ sensor and eddy covariance measurements, both approaches show similar circadian curves for the days 14–16 October 2016. The weak correlation could be explained by the fact that the G_btSP32L_ sensor has detected the actual sap flow according to the specific conditions where the tree SP32L is positioned, and the eddy covariance has reflected the *ET_EC_* encompassing an area with several trees.

Figure 11a is sectioned in three time intervals (13:12–18:57, 18:58–9:21, 9:22–23:45), aiming to analyse in detail the behaviour of the fluxes in each interval. The shaded interval (18:58–9:21) shows a consistent performance when compared to the low sap flow values in function of $k_{bt}$, given to be night-time. Conversely, the sap flow from $k_{cv}$ computes high values. The high *F_cv_* values are probably due to the fact that zero flow is not reached because of night-time water uptake for vegetative or reproductive growth and replenishment of internal storage [8]. The $k_{bt}$ approach showed to be useful to overcome the night-time sap flow measurement, adjusting $T_{\mathrm{heat}}$ and $T_{\mathrm{no}-\mathrm{heat}}$ values to $T_{MAX}$ and $T_{\infty }$.

The observations referring to the time interval (13:12–18:57) present a decrease in *F_bt_* values, ranging initially from 0.135 to 0.042 mm h^−1^, and at that point, increased up from 0.042 to 0.099 mm h^−1^. Similarly, to the time interval (9:22–23:45), a slight sap flow decrease occurred from 0.100 to 0.090 mm h^−1^, and at that point, increased up from 0.090 to 0.158 mm h^−1^. It could be explained as commented for the other vineyard in experiment II (Figure 10), where it is suggested that the tree uses a water-saving strategy assured by stomatal closure under drought stress in that daytime.

**Figure 12:**
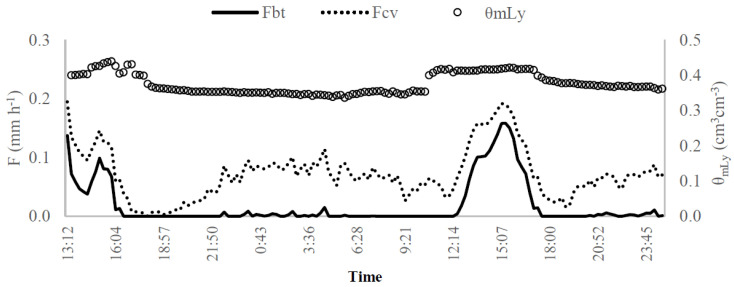
Circadian curve for days 14–15 October 2016 of soil moisture estimated with the mLy sensor (θ_mLy_, cm^3^ cm^−3^—empty circle: right vertical axis) and the transpiration (F, mm h^−1^—left vertical axis) estimates based on the sap flow conventional (dash line) and Biot-Granier (full line) approaches (Experiment III).

Figure 12 illustrates the circadian curves for the sap flow measurements with G_btSP32L_ sensor (*F_bt_*, mm h^−1^) and the soil moisture obtained with the weighting device (mLy) in days 14–16 October 2016. The soil moisture data (*θ_mLy_*, cm^3^ cm^−3^) obtained with mLy device showed to vary similarly to transpiration, showing the highest values simultaneously with the water redistribution into the soil, while the inverse occurs during the night. The explanation could be in the mLy device being subject under-canopy shading conditions. In [41], the authors suggested that the wind speed and the temperature variations within-canopy can affect energy and water balances since the canopy absorbs sensible heat from the soil, causing air temperature to decrease and a humid air accumulation. The humid air would be sourced from the high soil evaporation rate from the bare soil in days after rainfall events, verified in the day 287 (6.3 mm).

**Figure 13:**
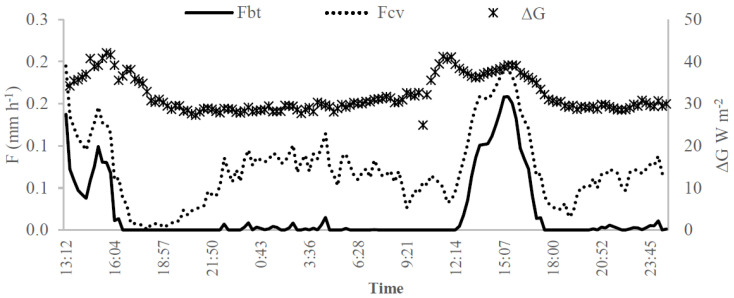
Circadian curves of the soil heat storage measured in days 14–15 October 2016 with the pair of Peltier cells (ΔG, W m^−2^—empty circle—right vertical axis) and the sap flow rate measurement (F, mm h^−1^—left vertical axis) with the Biot-Granier sensor set (SP32L) in function of the conventional (dash line) and Biot-Granier (full line) approaches (Experiment III).

Figure 13 illustrates the circadian curves for soil heat storage (ΔG, W m^−2^) and sap flow approaches *F_cv_* and *F_bt_* in the days 14–15 October 2016. It can be inferred that the variation of the temperature differences measured into the trunk is related to the soil heat storage in a similar way for *F_cv_* and *F_bt_* estimates. 

The pair of *Peltier* cells used to measure soil heat flux showed to be useful to fine-tune the sap flow estimates, given that the soil heat storage, soil water content and transpiration are intercorrelated. Thus, transpiration and soil water estimations can be checked for a low-cost and straightforward soil heat flux transductor.

## 4. Conclusions

The work presented herein addresses the evaluation of sensor prototypes and accuracy of a new sap flow measuring approach, the Biot-Granier sensing method (G_bt_), termed after being based on the verification of the Biot number and the well-known Granier method. For this purpose, sap flow measurements were performed using G_bt_ sensors placed in potted olives trees in a greenhouse and in two vineyards in open field experiments. 

Concerning the trial in the greenhouse with olive trees (Experiment I), transpiration measured with G_bt_ sensors showed to be compatible with the measurements observed with the gravimetric approach and those based on the Granier model, as well as being useful for sensors calibration. The use of an alternative Biot approach showed a significant improvement in sap flow estimations. The Biot-Granier sensors associated with Granier sap flow index computed based on the Biot number approach, showed the best conformity with the estimates of sap flow in comparison with the gravimetric test. Likewise, it showed to be useful to adjust the Granier sap flow index, a drawback of that methodology. Also, the sensitivity of the sensors reflected a satisfactory ability to detect the sudden sap flow reduction in water stress conditions. 

The empirical Granier equation transfers the temperature values to the sap flow index being thus strongly sensitive to the maximum temperature reached. Therefore, the temperature measurements from the sensor set should be related to each other because these measurements are a sensor-specific result, encompassing the characteristics of the sensor (installation, azimuth, sapwood around the sensor). The temperature measurements dynamically adjusted by the Biot-Granier approach demonstrated to be more appropriate, since these values allow decreasing the errors in the sap flow index computations. 

Regarding the field trials (i.e., Experiment II and III), the experimental studies showed that the Biot-Granier sap flow sensors provided good accuracy to measure temperatures in trees, to estimate the optimal ΔT_MAX_.

The Biot-Granier sensor prototypes were fully developed using easily obtainable parts. Their easy manufacturability and the inexpensiveness of the required components and materials makes them prone to be fabricated at low cost and thus widely adopted. Also, that technique is addressed for public domain, which implies that all the creative work holds not exclusive intellectual property rights. The same can be said regarding the data acquisition and control hardware, but this requires a more electronics oriented specific knowledge. This fact, together with the reasonable accuracy of the results that were obtained in the lab and field experiments carried out in two orchards, confirm the proposed customizable solution as one to obtain cheaper, more flexible and accurate sap flow sensors.

Future research should focus more strongly on validating the Biot-Granier approach for an extended period. In this first phase, priority has been given to analysing the thermodynamic processes in general. In a second phase, considering that the technical characteristics of the Biot-Granier sensor will be consolidated, the new goal will be to repeat the experiment with a more robust statistical plan.

## Figures and Tables

**Table 1:** Technical comparison between the original *Granier* sap flow and the Biot-Granier sensor.

Criteria	Conventional *Granier* Approach (Gcv)	Biot-Granier Approach (Gbt)
Sensing component	Type T Thermocouple (Copper/Constantan)	Thermistor
	Wide range of temperature sensing (−200 °C to 350 °C)	Narrow range of sensing (−55 °C to 150 °C)
	Requires a high-resolution measuring system such as a voltmeter to measure the output voltage.	Requires an external voltage to operate as a measuring device.
	Difficult construction.	Easy construction.
	The voltage generated at different temperatures is relatively low (around 43 µV/°C). Thus, amplification is required.	Resistance based measurement process—a voltage divider set with a reference resistor tied to a reference voltage usually is enough. Thus, amplification is not required.
Heating component	Constantan Wire (range 14 to 20 Ω)	Nickel-chrome Wire (range 30 to 40 Ω)
**Operational Features**		
**Type of Measurement**	**Differential Temperature**	**Absolute Temperature**
Temperature sensing location	Inner of the heating tube.	Outer of heating tube, on the needle tip.

**Table 2:** Description of the diameter trunk and sensor localisation on the tree.

Olive Trees	^1^ A_TK_ (cm^2^)	Azimuth	^2^ h|s (cm)	^3^ p|s (cm)
P01	24	N	26	86
P02	18	N	37	120
C01	21	N	30	100
C02	21	N	40	120

^1^ A_TK_: Trunk area; ^2^ h|s: distance between T_heat_ and soil surface; ^3^ p|s: height of the tree from top to soil surface.

**Table 3:** Description of the experiments using the Biot-Granier sap flow sensors.

^1^ Exp	Description	Period (Days of Year)	Objective	Sensor Sets
I	Potted olive trees in a greenhouse (variety “Galega”)	69 to 110 (2018)	comparison with the gravimetric method and commercial sap flow sensors in the greenhouse	P01, P02, C01, C02
II	Vineyard (variety “Tempranillo”)	201 to 240 (2017)	comparison with commercial sap flow sensors in the experimental field	SP04, Gr3
III	Vineyard (variety “Galego Dourado”)	245 to 305 (2016)	comparison with eddy covariance, soil water content and soil heat flux	SP32L

^1^ Exp: Experiment number.
